# Effectiveness of Radial Extracorporeal Shock Wave Therapy and Visual Feedback Balance Training on Lower Limb Post-Stroke Spasticity, Trunk Performance, and Balance: A Randomized Controlled Trial

**DOI:** 10.3390/jcm11010147

**Published:** 2021-12-28

**Authors:** Emanuela Elena Mihai, Ilie Valentin Mihai, Mihai Berteanu

**Affiliations:** 1 Physical and Rehabilitation Medicine Department, Carol Davila University of Medicine and Pharmacy Bucharest, 050451 Bucharest, Romania; emanuela-elena.mihai@drd.umfcd.ro (E.E.M.); mihai.berteanu@umfcd.ro (M.B.); 2 Department of Telecommunications, University Politehnica of Bucharest, 060042 Bucharest, Romania; 3 Physical and Rehabilitation Medicine Department, Elias University Emergency Hospital, 011461 Bucharest, Romania

**Keywords:** radial extracorporeal shock wave therapy, stroke, spasticity, balance trainer, stabilometric assessment, neurological rehabilitation

## Abstract

Stroke remains one of the leading causes of disability in adults, and lower limb spasticity, affected stance, and balance impact everyday life and activities of such patients. Robotic therapy and assessment are becoming important tools to clinical evaluation for post-stroke rehabilitation. The aim of this study was to determine in a more objective manner the effects of visual feedback balance training through a balance trainer system and radial extracorporeal shock wave therapy (rESWT), along with conventional physiotherapy, on lower limb post-stroke spasticity, trunk control, and static and dynamic balance through clinical and stabilometric assessment. The study was designed as a randomized controlled trial. The experimental group underwent conventional physiotherapy, visual feedback balance training, and rESWT. The control group underwent conventional physiotherapy, visual feedback training and sham rESWT. The statistical analysis was performed using GraphPad Software and MATLAB. Primary clinical outcome measures were The Modified Ashworth Scale (MAS), passive range of motion (PROM), Visual Analogue Scale (VAS), and Clonus score. Secondary outcome measures were trunk performance, sensorimotor, and lower limb function. Stabilometric outcome measures were trunk control, static balance, and dynamic balance. Visual feedback training using the Prokin system and rESWT intervention, along with conventional physiotherapy, yielded statistically significant improvement both on clinical and stabilometric outcome measures, enhancing static and dynamic balance, trunk performance, sensorimotor outcome, and limb function and considerably diminishing lower limb spasticity, pain intensity, and clonus score in the experimental group.

## 1. Introduction

Stroke remains one of the leading causes of death and disability all over the world [1,2,3]. Trunk control and sitting balance are commonly affected in post-stroke patients and are considered important features to predict functional outcome and hospital stay [4,5,6,7,8,9]. Moreover, balance and gait impairments are known to highly interfere within the recovery process of standing and walking ability in post-stroke patients [10,11,12]. Another important characteristic in many post-stroke patients is spasticity, along with muscle weakness, sensorimotor deficits, and cognitive impairments [13,14,15]. In addition, trunk impairment, spasticity grade, poor balance, and altered stance in post-stroke patients are increasing the risk for falls and impaired mobility [14].

Trunk deviations may also imply balance deficit, gait impairment, and diminished functional ability, but core stability exercises may also provide more efficient use of the lower limbs for static and dynamic balance, as well as for gait [7,15,16,17,18]. Given that, in the acute phase of stroke, trunk function is a major indicator for functional independence outcome, clinical assessments for trunk deviations and lower extremity tests are both used, predicting independent walking at six months [19,20]. To ensure an adapted rehabilitation program and assess the main determinant, it is important to differentiate the intrinsic trunk control deficiency from underlying lower extremity deficits. Nevertheless, to our knowledge, it has not yet been evaluated, nor determined, to what extent trunk deficits and lower limb post-stroke spasticity may correlate and impact stance and balance in post-stroke patients. Therefore, the aim of our study was to assess, in a more objective, global manner, both clinically and through stabilometric system Prokin, the relationship between stance, balance, trunk deviations, and lower limb post-stroke spasticity. Moreover, the trial aimed to determine whether conventional physical therapy in conjunction with visual feedback balance training and two radial extracorporeal shock wave therapy (rESWT) sessions might have a positive impact on stance, balance, and spasticity grade, and we assessed their relationship with trunk performance in post-stroke patients. Additional primary and secondary outcome measures were also evaluated. Core stability exercises and visual feedback training were used, with promising results [4,10,16,21,22]. The stabilometric computerized system is both an assessment tool and a training system for static, dynamic balance, and trunk. To date, it was used in various clinical trials as a training tool for promoting stance and balance improvement in several neurorehabilitation programs [10,23,24].

For spasticity management, along with physical therapy programs, anti-spastic medications or botulinum toxin type A injections, selective neurotomy, chemical neurolysis, and orthopedic surgery, techniques, such as whole body vibration, local muscle vibration, neuromuscular electrical stimulation (NMES), and therapeutic ultrasound, were also used, but there is no clear consensus yet regarding their long-term effects, number or sessions, or duration of treatment [25]. The whole body vibration seemed to reduce lower limb spasticity in cerebral palsy patients, and local muscle vibration therapy granted effectiveness in the management of chronic post-stroke patients [26,27]. In addition, NMES alone or combined with other interventions showed beneficial effects on lower limb motor function in chronic stroke patients [28]. Therapeutic ultrasound seemed to have good efficacy when also compared to extracorporeal shock wave therapy (ESWT) [29]. During the last few years, extracorporeal shock wave therapy, a novel, non-invasive intervention has become a potential therapy in the non-invasive management of post-stroke spasticity, with promising results also on the long-term [29,30,31,32]. The principle of this therapeutic intervention involves a sequence of an acoustic pulse producing high peak pressure, fast rise in pressure, and short time cycle targeting the wanted area and providing effective outcomes [29,33]. The mechanism of propagation of the shock wave refers either to radial extracorporeal shock wave therapy (rESWT) or focused extracorporeal shock wave therapy (fESWT). rESWT actions on a larger interventional area and delivers the high energy in the superficial tissue, modifying globally the mechanical properties of the muscle, in comparison to fESWT, where the shock wave action is delivered to a selected, deeper intervention area [29,30,31]. 

Considering the rESWT mechanism of action and its positive results from the literature with regard to spasticity, we aimed to assess rESWT effects in conjunction with conventional physiotherapy and balance training for post-stroke patients affected by lower limb spasticity. Therefore, we conducted a randomized controlled trial to further investigate and assess the relationship between lower limb spasticity and trunk deficits during static and dynamic balance in post-stroke patients who underwent either rESWT or sham rESWT intervention, visual feedback balance training using the Prokin system, and conventional physical therapy. The stabilometric assessment complemented the clinical evaluation and also provided an objective evaluation of combined therapeutic effect of the used therapies. Additional outcomes focused on the effectiveness of rESWT delivery on pain intensity, clonus, passive range of motion, lower limb sensorimotor function, and functionality. The adverse events were also attentively monitored during the trial.

## 2. Materials and Methods

### 2.1. Ethical Approval

The present study was conducted according to the Declaration of Helsinki, the guidelines for Consolidated Standards of Reporting Trials (CONSORT) and the CONSORT Statement [34]. The study protocol was approved by the Ethics Committee of the Elias University Emergency Hospital, Bucharest, Romania, Prot. No. 2090/C.E. Written informed consent was received from all participants taking part in the study, as well as from their relatives, when necessary.

### 2.2. Study Design

The study is designed as a prospective, double-blind randomized controlled trial, single-center, with an intention-to-treat analysis, and two groups with 1:1 allocation ratio. Patients were recruited between January 2021 and August 2021 during the pandemic COVID-19 period and were randomly allocated either to a control or an experimental group. The allocation method was concealed in numbered, opaque, sealed envelopes. The recruitment of patients, conventional rehabilitation program, intervention delivery, and data collection were performed at the Physical and Rehabilitation Medicine Department, Elias University Emergency Hospital, Bucharest, Romania.

### 2.3. Study Participants

A total of 273 patients admitted to the Physical and Rehabilitation Medicine Department, Elias University Emergency Hospital, Bucharest, Romania, between January 2021 and August 2021, were evaluated for eligibility, and 39 patients met the eligibility criteria. Seven participants declined to take part in the study (*n* = 7), and four participants were not able to initiate the rehabilitation program because of interfering acute diseases (*n* = 4), other complications (*n* = 4). One patient was not able to complete the rehabilitation program due to early discharge related to personal and family matters (*n* = 1). The final analysis and results are based on 23 patients with post-stroke lower limb spasticity who were enrolled and completed the study. No significant differences were found at the baseline comparison. Post-treatment data for the patients not completing the study were not available.

Patients with stroke were included if they stated the following inclusion criteria: (1) a hemorrhagic or ischemic stroke in acute, subacute, or chronic phase; (2) no history of previous stroke; (3) lower limb post-stroke spasticity and spasticity grade ≥ 1 on the Modified Ashworth Scale (MAS); (4) pain intensity measured on Visual Analogue Scale (VAS) ≥ 1; (5) ability to stand unassisted in upright position for 30 s; (6) no change in anti-spastic drug dose or treatment, and no changes in analgesic medication, as it could affect the results on the Modified Ashworth Scale and the Visual Analogue Scale; and (7) adult patients (>18 years old). Exclusion criteria consisted of: (1) other neurological and orthopedic disorders or lower limb deformities that could interfere with motor performance and balance; (2) myopathies; (3) severe cognitive impairment, severe aphasia or inability to understand instructions; (4) severe spasticity; (5) visual field conditions or hemineglect; (6) patients unable to undergo follow-up evaluation and excluded from the final analysis; and (7) anticoagulant medication or any contraindication to receive rESWT, or any contraindication to physical therapy. Informed consent was obtained from all the patients prior to their participation, and the study was approved by the hospital’s Ethics Committee according to the Declaration of Helsinki.

In the study and final analysis, there were enrolled 13 male patients and 10 female patients, with an average age of 68.18 ± 11.51 years old in the control group and 60.33 ± 11.5 in the experimental group. The average duration of disease was of 24.97 ± 34.17 months in the control group and 25.02 ± 39.23 in the experimental group. There were 17 cases diagnosed with ischemic stroke based on cerebral MRI or cerebral CT scan, and 6 cases diagnosed with hemorrhagic stroke.

### 2.4. Radial Extracorporeal Shock Wave Therapy (rESWT) Intervention

Conventional physiotherapy could be defined as the therapeutic interventions and techniques carried out in accordance to each rehabilitation department. In our study, we referred to it as a treatment involving any of the following practices and therapies for post-stroke patients (range of motion exercises, stretching, stance and balance training, core stability exercises, gait training, functional training, physical agents and other therapies, orthoses, braces, etc.). Both groups had the same conventional physical therapy program, consisting of techniques of verticalization, passive and active movements, stretching exercises, stance and balance techniques, gait training, functional training, therapeutic massage, local heat, or cryotherapy. The conventional physiotherapy program lasted 1 h/day, 5 days/week, for 2 weeks. A summary of the parameters for the control group and the experimental group is presented in Table 1.

After performing the baseline screening and assessment, random allocation was used to generate allocation sequence and assign the patients to one of the two groups. Patients considered eligible were assigned to an experimental group (receiving conventional physiotherapy, visual feedback balance training using the Prokin and rESWT) or a control group (receiving conventional physiotherapy, visual feedback balance training using the Prokin and sham rESWT). Treatment allocation was concealed from all the participants and the outcome assessors during the trial. 

**Figure 1:**
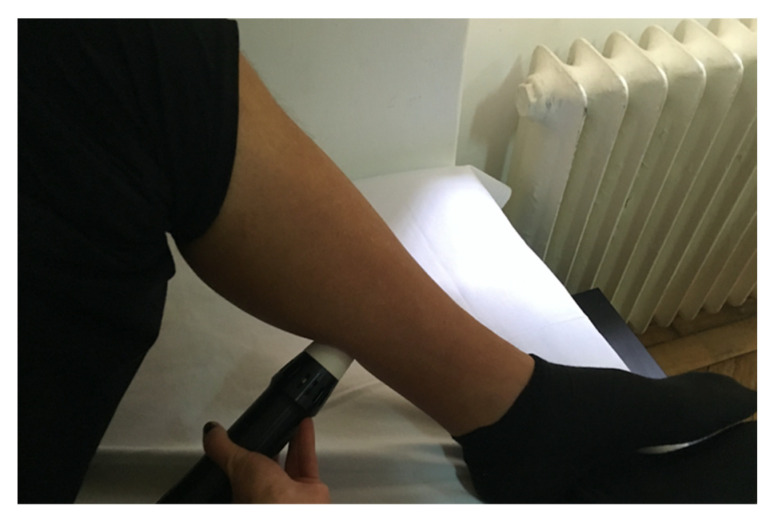
Radial extracorporeal shock wave therapy delivery on a patient from the clinical trial; original picture taken by the author EE Mihai in the Department of Physical and Rehabilitation Medicine, Elias University Emergency Hospital, Bucharest, using a digital camera (Nikon D3300; Nikon Corporation, Tokyo, Japan).

After choosing the intervention site, rESWT (Endopuls 811; Enraf Nonius B.V. Vareseweg 127, 3047 AT Rotterdam MedTech, The Netherlands) was applied at the myotendinous junction of both the gastrocnemius and the soleus muscles in post-stroke spasticity patients. No ultrasonography was used to detect the intervention site. All the patients completed two rESWT sessions once a week for two weeks. Participants were comfortably placed during the rESWT intervention, and 2000 shots were applied on the gastrocnemius and soleus myotendinous junction with a frequency of 10 Hz and energy density of 60 mJ (1 bar), within tolerable pain limits. The control group received sound over the myotendinous junction of gastrocnemius and soleus with transducer-like contact. Treatment sessions lasted for approximately 7 min each, 2 sessions in total, 1 session/week, for 2 weeks, for sham rESWT and rESWT. The rESWT and sham rESWT sessions were delivered during the hospital stay, 15 min after the conventional rehabilitation program and the visual feedback balance training. Figure 1 presents the rESWT delivery.

Clinical and stabilometric assessments were performed before the first rESWT session and the beginning of physical therapy program. The second evaluation was performed at the end of the rehabilitation program, after the last session of treatment by the same assessor, who also performed the first assessment. Adverse events were attentively monitored during the study.

### 2.5. Visual Feedback Balance Training Using the Prokin

**Figure 2:**
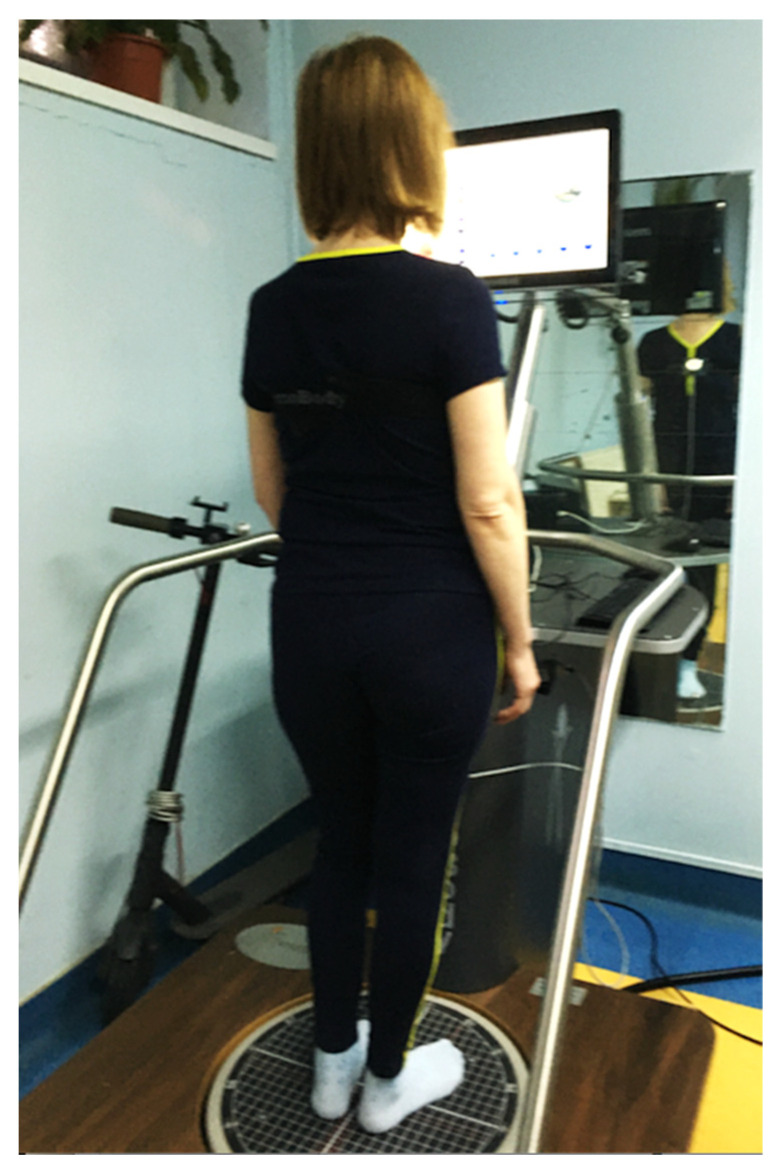
Patient from the clinical trial during a session of visual feedback balance training and stabilometric assessment using the Prokin system; original picture taken by the author EE Mihai in the Department of Physical and Rehabilitation Medicine, Elias University Emergency Hospital, Bucharest, using a digital camera (Nikon D3300; Nikon Corporation, Tokyo, Japan).

All the patients in the experimental and the control group performed visual feedback balance training using Prokin, in addition to the conventional rehabilitation program, rESWT intervention, and sham rESWT, respectively. They were given precise information, and they were asked to wear normal clothing in order to create a more friendly environment during the session. Each training session lasted for 20 min/day, 5 times/week, for 2 weeks, and was delivered 15 min after the conventional physiotherapy program, as post-stroke patients are usually becoming easily fatigued. Through real-time visual feedback, patients were asked to move their Center of Pressure (CoP) in different directions, maintaining the specified area on the screen. They had to move forward, backward, sideways, and to perform a circular motion. For trunk training, they had to perform the same movements while they were seated on the specific Prokin trunk sensor. The patients were also given the possibility to play one game of their choice at the end of the training. Figure 2 shows one of the patients on the stabilometric system, Prokin balance trainer, and assessment tool.

### 2.6. Clinical Outcome Measures and Methods of Clinical Evaluation

The primary clinical outcome measures were spasticity grade assessed using the Modified Ashworth Scale (MAS), knee and ankle passive range of motion (PROM), pain intensity evaluated through Visual Analogue Scale (VAS), and Clonus score. For the MAS evaluation for gastrocnemius and soleus muscles, patients were laying in supine position with the knee fully extended and the joint stabilized. The MAS measures muscle resistance during passive muscle stretching. The MAS score ranges from 0 (no increase in muscle tone) to 4 (rigid) and includes a rating of 1+. For a suitable statistical analysis, a MAS grade 1+ was matched to 2 points and grade 2, 3, and 4 were matched to 3, 4, and 5 points, respectively. Knee and ankle passive mobility were assessed by PROM, using a hand-held goniometer, and summing the angles of maximum plantar flexion and dorsiflexion. Pain intensity was evaluated by the VAS using a vertical 10-cm line (starting pain-free to the worst imaginable pain). The Clonus score evaluated the beats’ number up to sustained clonus.

The secondary outcomes were gait and balance assessed by Tinetti Assessment Tool, Functional Ambulation Categories (FAC), and Fugl-Meyer Assessment for Lower Extremity (FMA-LE). FMA-LE assessed lower limb sensorimotor impairment, and Trunk Impairment Scale (TIS) evaluated static and dynamic sitting balance and trunk coordination in a sitting position. The functional impairments were assessed through Tinetti Assessment Tool and FAC. The outcomes were classified in relation to the baseline (T0) and the follow-up period after two sessions of rESWT intervention or sham rESWT intervention(T1), and between the control and the experimental group. ESWT was applied once a week for two weeks, and the assessment was performed pre-treatment and after the second session. The assessment was conducted at baseline and follow-up by the same blind assessor, who did not know which patients underwent rESWT or sham rESWT.

### 2.7. Stabilometric Assessment

The stabilometric assessment was performed by a blind assessor using the Prokin system (PK 252, TecnoBody, Bergamo, Italy), and the assessor did not know which patients underwent rESWT or sham rESWT. Prokin system consists of a force-sensitive stabilometric platform which assists with the assessment of trunk, stance, and static and dynamic balance. Additionally to clinical measures, we aimed to evaluate stance and balance in an objective manner, while correlating the clinical assessment with the data obtained using the Prokin system. Focusing on an objective approach and trying to recreate a daily life environment, the patients were asked to wear comfortable clothing. They were given precise instructions and were reminded of them when needed; they were positioned in a standardized way on the stabilometric platform (barefoot or wearing socks, but no footwear), and the feet position was adapted by using a Y shaped frame (called yova) with a 3-cm distance between the internal malleoli. In addition, the medial border of the feet was rotated 12 degrees in correlation with the anteroposterior axis. Patients were instructed to stand still, keep their arms by their sides, and look ahead at the screen positioned in front of them focusing on a stationary or moving target. Each participant performed various tests in standing position and also in seated position, allowing the evaluation of stance, balance, and trunk. In addition, the tests were performed either with eyes open (EO) or eyes closed (EC), and a skilled physiotherapist stood behind the patient to prevent any risk of falls. The perimeter and the ellipse area for static balance, dynamic balance, and trunk parameters were measured, and results were evaluated accordingly.

Clinical evaluation and stabilometric assessment using the Prokin system at baseline and follow-up were both performed in two sessions. Conventional rehabilitation program, visual feedback balance training using the Prokin, and rESWT or sham rESWT intervention, clinical evaluation, and instrumental assessment were performed, and patients were asked to use the same comfortable clothing, the same conditions were recreated and instructions were once again given to all the participants. For both the control group and experimental group, none of the patients experienced loss of balance during stabilometric assessment in any of the sensory conditions. None of the patients suffered any accident or fall during the assessments and study duration, physiotherapy program, nor during hospitalization. In addition, no adverse events related to rESWT intervention were reported during or after sessions. The stabilometric assessment using the Prokin platform was carried out by the same experienced physiotherapist, who was not involved in the screening, randomization sequence, baseline evaluation, and follow-up.

### 2.8. Statistical Analysis

The assessment of all the patients was conducted before and after the intervention. Patient characteristics were described as the mean and standard deviation for the control and the experimental group for the continuous data. The Pearson’s Chi-square test was used when analyzing differences in categorical variables, and the Mann–Whitney U test and Independent *t*-test were used for continuous variables. The change score was also used to show the difference between the pre- and post-treatment, both for clinical outcome measures and stabilometric outcome measures. The change in outcome measures was compared between the control and the experimental group by means of the Mann–Whitney U test and Independent *t*-test.

All the statistical analyses were performed using GraphPad Software (San Diego, CA, USA) and MATLAB (R2016a, The MathWorks, Inc., Natick, MA, USA) to determine the variables of interest. The null hypothesis was rejected when the critical test statistical value α was exceeded by the F value/t value. A *p*-value < 0.05 was considered to be statistically significant.

## 3. Results

**Figure 3:**
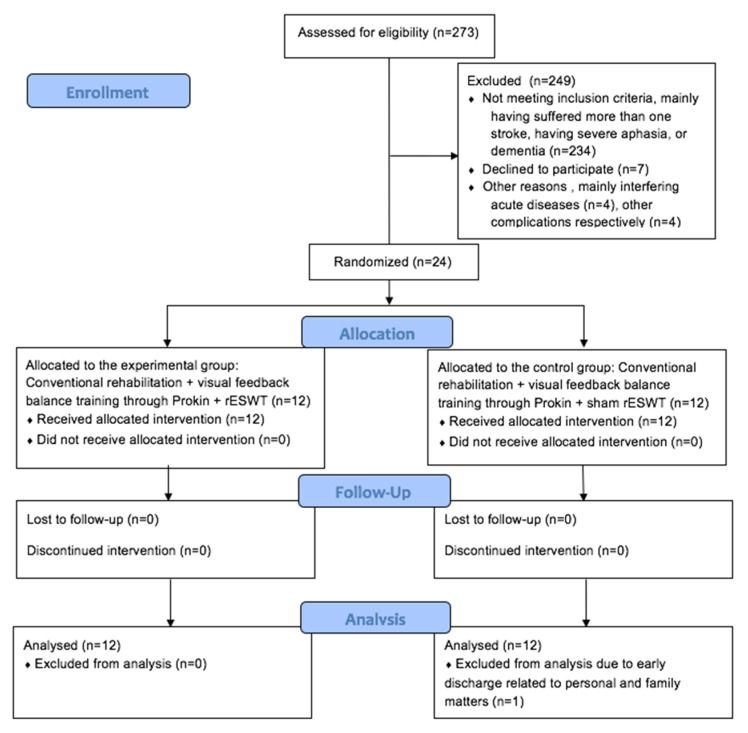
CONSORT 2010 Flow Diagram and patient allocation. Abbreviation: rESWT: radial extracorporeal shock wave therapy.

Figure 3 shows the study flow diagram and patient allocation. Out of the 273 participants screened, 24 patients were randomly assigned, with 12 patients either in the control group or the experimental group. One patient in the control group was discharged earlier due to personal matters and was excluded from the analysis. Therefore, the patients were randomized into two groups: a control group (*n* = 11) and an experimental group (*n* = 12). 

Table 2 presents the characteristics of control and experimental group at baseline. No differences were identified among the groups related to the demographic variables, stroke type, affected side of the body, time since stroke onset, nor clinical and stabilometric outcomes. Comparisons between the groups showed homogeneity at baseline for all the parameters.

### 3.1. Clinical Outcome Measures

Table 3 presents the comparison of the intervention effect among control and experimental group for primary and secondary clinical outcome measures. *p*-value shows the inter-group differences of the change score. For the primary and secondary clinical outcome measures, patients from the experimental group showed significant improvement compared to the control group in all the outcome measures except for knee PROM, TIS, and FAC.

The change score for the MAS was 1.08 in the experimental group, while, in the control group, it was 0.36, (*p*-value < 0.02). For ankle PROM, a statistically significant improvement was also stated, with a change score of 8.17 in the experimental group and 4.82 in the control group, (*p*-value < 0.03).

For the pain intensity assessment through VAS, the change score was 2 points in the experimental group, while the change score in the control group was 1 point, showing a statistically significant difference between the two groups after the intervention, (*p*-value of 0.02). Regarding the Clonus score, the score in the control group was 0.46 points, while, in the experimental group, it was 1.42 points (*p*-value < 0.01).

As for the other parameters, such as Tinetti Assessment Tool, the experimental group showed a change score of 9.83 points compared to the control group, which showed a change of 5.28 points (*p*-value < 0.02). With regard to the FMA-LE, the experimental group scored 5.09 points in the change score, and the control group registered 3.09 points (*p*-value < 0.01).

### 3.2. Stabilometric Outcome Measures

The stabilometric outcome measures evaluated using the Prokin system after the intervention are presented in Table 4. All the parameters showed statistically significant improvement, except for perimeter with eyes closed (EC). However, before the intervention, there were no differences between the control and the experimental group. Regarding the dynamic balance, the experimental group showed a change score of 2.17 compared to the control group, which registered a change score of 0.35 (*p*-value < 0.03). As for limits of stability testing, there was a score change of 265.64 points in the experimental group compared to the control group, where the change score was 109.04 points (*p*-value < 0.02).

Concerning the trunk analysis, a statistically significant improvement was found (*p*-value < 0.02) in the experimental group, with a change score of 156.6. Regarding the static balance, the assessment of perimeter with eyes open (EO), ellipse area with EO, and ellipse area with EC showed that scores were significantly decreased after conventional physical therapy, Prokin visual feedback balance training, and rESWT intervention (*p*-value < 0.05) in the experimental group, compared to the control group.

**Figure 4:**
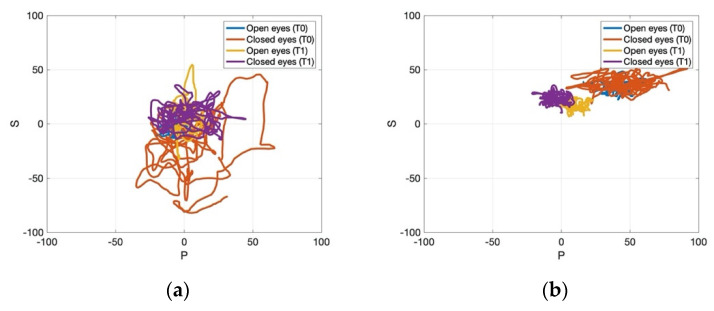
Static stabilometric assessment with eyes open and eyes closed for one representative patient in the control group (**a**) and experimental group (**b**) pre- and post-treatment. Abbreviations: T0: pre-treatment; T1: post-treatment.

**Figure 5:**
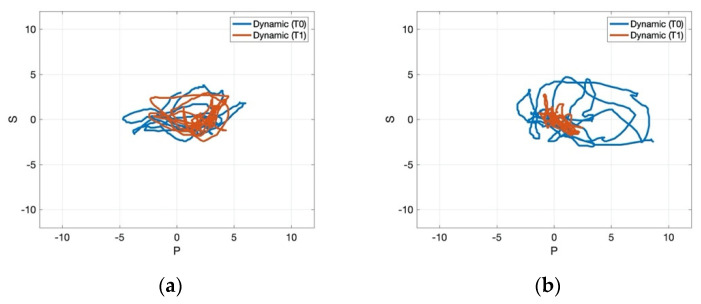
Dynamic stabilometric assessment for one representative patient in the control group (**a**) and experimental group (**b**) pre- and post-treatment. Abbreviations: T0: pre-treatment; T1: post-treatment.

**Figure 6:**
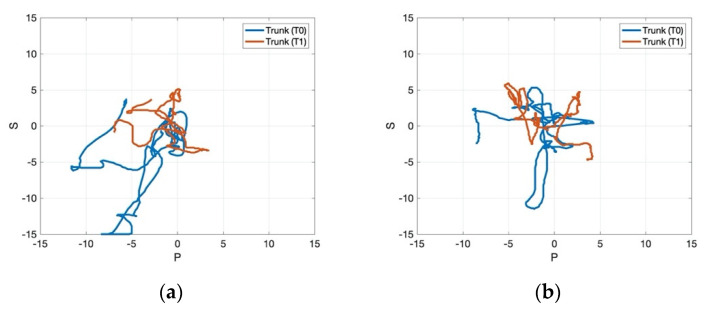
Trunk stabilometric assessment for one representative patient in the control group (**a**) and experimental group (**b**) pre- and post-treatment. Abbreviations: T0: pre-treatment; T1: post-treatment.

Figure 4, Figure 5 and Figure 6 present the data processed through MATLAB for one representative patient from the control and the experimental group pre- and post-treatment. Figure 4 showed that the experimental group gained a more pronounced improvement regarding the path recorded by the CoP for 30 s, which became more stable after the application of rESWT, visual feedback balance training, and conventional physiotherapy for the static stabilometric assessment. Regarding the dynamic stabilometric assessment, Figure 5 showed a smoother CoP path and a reduction of CoP path length for the experimental group after the intervention and, therefore, improvements for stance and dynamic balance. Figure 6 showed the processed data from trunk stabilometric assessment for trunk stability in the experimental group compared to the control group.

No adverse events, such as muscle hematoma, focal edema, pain, or skin petechiae, were reported during the study. No falls were registered, either, and all patients were assessed according to the study protocol.

## 4. Discussion

Post-stroke patients are often affected by limb spasticity, strength deficits, loss of function, loss of balance, and trunk deficits. Accordingly, novel interventions could complement conventional rehabilitation and common medications used in neurorehabilitation with satisfying results. This study’s results showed that additional visual feedback training using the Prokin and rESWT intervention improved not only clinical parameters, but also stabilometric parameters in the experimental group. Since the control group received sham rESWT, and the results were less significant than in the experimental group, rESWT was considered the main determinant, especially for the clinical parameters. An important feature of the study is the objective, global assessment of post-stroke patients through clinical and stabilometric parameters. In our trial, Prokin was used as a training tool, as well as an assessment instrument.

To our knowledge, this is the first trial to assess, clinically and through stabilometric parameters, the effects of both visual feedback training using the Prokin system and rESWT intervention added to conventional physiotherapy for post-stroke survivors. In addition, we expanded the trial on correlating trunk deficits with lower limb spasticity and the cumulative effects of combined therapies on clinical and stabilometric outcome measures. 

The visual feedback balance training and rESWT intervention added to conventional physiotherapy were implemented aiming to improve stance, balance, trunk deficits, and to decrease lower limb spasticity grade. The results are consistent with previous studies in which the main focus was either on core stability exercises, trunk training, or visual feedback training [4,10,14]. Virtual reality added to conventional rehabilitation also showed efficacy on improving balance and gait in neurologic patients [35,36]. As an additional treatment technique to the conventional physiotherapy, visual feedback balance training using the Prokin system has gained more interest since it enables patients to adjust their stance, balance, and overall performance in real-time through visual dynamic feedback. Therefore, patients were able to adapt the movement of the CoP and redress an abnormal stance, consequently improving balance function. This training system offers a novel, interactive tool as an alternative to sole conventional therapy. The training using the Prokin system ensures the projection of a more accurate proprioceptive sensing map, which helps the patient focus on regaining proprioceptive function, as well as to adjust more easily to sensorimotor perturbations, and this could also explain the beneficial effects on static and dynamic balance that we obtained in our study.

In our study, the experimental group showed a statistically significant change of the stabilometric outcome measures compared to the control group. Both static and dynamic balance were improved, which could also be correlated to the Tinetti Assessment Tool score through Pearson correlation analysis, obtaining clinical and stabilometric enhancement (*p*-value < 0.02). The dynamic module allowed the assessment and management of instability by the patient, offering real-time insights into adjusting mechanisms, thus allowing the patient to redress the stance, and, consequently, the balance, as well. The static module assessed the patients through the oscillation of the CoP, area, and the parameter of the CoP. For the experimental group, in the EO situation, area, as well as perimeter, decreased dramatically. A possible explanation could be that, after rESWT intervention the spasticity grade affecting the lower limb decreased, and the proprioceptive training was also performed more easily, leading to the smoothness of oscillation and a more stable CoP path. Moreover, these improvements may offer more information regarding the relationship between lower limb spasticity, trunk deficits, and balance. Another possible explanation could be related to the visual compensation, which can overcome the shortcomings of proprioceptive and vestibular function deficiency, enhancing neuroplasticity and, therefore, treatment efficacy. The only stabilometric parameter which showed no statistically significant change was perimeter in the EC situation.

The limits of stability assessed the participants’ ability to voluntarily sway to various locations in space, as shown on the screen while tested. The measured parameters were maximum CoP excursion, endpoint CoP excursion, and directional control. After the intervention, both groups showed enhanced results, but the experimental group showed three times better results compared to the control group, showing that the rESWT component determined the more enhanced effect. The trunk module allowed the assessment of the pelvic area in the sitting position, and the trunk sensor detected oscillations of the torso in every direction. It offered information on peripheral control of the patient and possible compensation for poor stance. Compensation strategies are factors which are usually seen in post-stroke patients and sometimes interfere within the rehabilitation programs. Balance control is a key feature associated with gait recovery and the probability of suffering a fall in stroke patients, but adapted strategies and individualized rehabilitation programs augment therapeutic efficacy in the long-term [10,37,38,39]. Regarding trunk control, the stabilometric assessment showed a more pronounced effectiveness on this parameter in the experimental group compared to the control group. The clinical parameters, such as spasticity grade, clonus score, and passive range of motion, were also significantly enhanced for the group receiving rESWT, compared to the group receiving sham rESWT. These results may explain how adequate trunk control could lead to a smoother, more stable center of pressure path and, therefore, better static and dynamic balance. In addition, improved lower limb function could also enhance a more correct stance and balance.

Concerning spasticity grade assessed by the MAS, in one study, significant differences were found between the control and the experimental group, but a change score of one point was required for considering a detectable clinical change in stroke participants [15]. However, it was also observed that there was a pronounced tendency for the patients experiencing a more severe lower limb impairment to have more important trunk movement deviations, which could be related to the stability ensured by lower extremity [15]. Although, in our study, we found a change score of 0.68 points between the control and the experimental group, the change score in the experimental group post-treatment was 1.08 points and 0.36 points, respectively, in the control group. In our trial, patients with a higher spasticity grade also experienced more trunk deficits, and the intervention effect showed significant improvement of these parameters, both on clinical and stabilometric evaluations. In contrast to what was observed in another study, the lower limb spasticity grade improvements could not only be associated with transitory factors, since improvement in lower limbs spasticity had a significantly positive impact on stance and balance at the clinical and stabilometric evaluations and performance [4]. Accordingly, the relationship between lower limb spasticity grade and trunk deficits cannot be excluded and should be further analyzed.

The clinical improvement of MAS grade was three times greater in the experimental group compared to the control group showing the effectiveness of the rESWT component on spasticity management and also the dynamic real-time feedback through balance training. These results are consist with those from other clinical trials [10,29,40]. Although knee PROM showed no statistically significant improvement after the interventions, ankle PROM showed statistically significant improvement, which could be explained by the site of rESWT application and its effects on plantar flexor muscles and thus, on augmented ankle PROM. Another explanation for more significant improvement of ankle PROM may be related to the effects of rESWT on the entire muscle and the superficial tissue area. These results are in accordance with other studies [30,40]. In a previous study, rESWT seemed to provide greater improvement in ankle PROM than fESWT [29]. The difference between rESWT and fESWT lies in the penetration depth and physical properties, but the clinical difference is not yet determined [29]. However, to assess the effect on the muscle, reliable measurements of muscle thickness on ultrasonography are correlated with the time frame, as well as the side of the body [41]. Several studies have already showed that both rESWT and fESWT were effective as novel, non-invasive therapies for spasticity management in post-stroke patients [29,31,40]. In terms of lower limb pain intensity, the VAS showed better scores in the experimental group, twice as ameliorated than in the control group which received sham rESWT and Prokin visual feedback training, the results being consistent with another trial which also showed the beneficial effects of rESWT on this endpoint [42]. The effects on pain intensity could be explained through the properties of extracorporeal shock waves on the muscles and tendons, which was found to produce a long-term tissue regeneration effect, as well as a prompt antalgic and anti-inflammatory result [31,43,44]. No improvement was found for the TIS and FAC, although TIS was significantly improved in the experimental group. An explanation could be that many patients were already in the chronic phase of stroke, and, although the clinical improvement was registered, it was not found as statistically significant. Both Tinetti Assessment Tool and FMA-LE showed amelioration, demonstrating statistically and clinically significant sensorimotor and functional improvement. These parameters were also correlated with the stabilometric outcome measures.

The present study also has some limitations. Firstly, the small sample size could augment the overall effect of the intervention. However, correlations were performed, and all the clinical and stabilometric parameters were cautiously evaluated and measured. Secondly, study data might be limited since this is a single center trial. Nonetheless, this could provide more accurate data from groups assessed by the same team in the same medical unit and under the same circumstances. Thirdly, patients who could not tolerate the upright position for at least 30 s could not be eligible to take part in our trial, which limited the number of participants. Therefore, future research and larger samples of participants are highly needed to assess the efficacy of visual feedback balance training using the Prokin system combined with rESWT and conventional physiotherapy program in treating post-stroke patients, lower limb spasticity, and its relationship with trunk deficits and static and dynamic balance.

Despite these limitations, the present study has proved that combined rESWT intervention and visual feedback training, along with conventional physiotherapy, yielded statistically significant improvement, both on clinical and stabilometric outcome measures, enhancing static and dynamic balance, trunk performance, sensorimotor outcome, and limb function and considerably diminishing lower limb spasticity, pain intensity, and clonus score in the experimental group. Our results could also explain the relationship between spasticity, trunk deficits, and poor balance, as well as the way they influence each other. 

## 5. Conclusions

In conclusion, rESWT intervention and visual feedback balance training using the Prokin system, along with conventional physiotherapy, improved trunk control and lower limb spasticity, and static and dynamic balance, decreased pain intensity and clonus score, and ameliorated the sensorimotor outcome and functionality in post-stroke patients. These results need to be further confirmed by larger clinical trials, and future research should further assess the effects of additional therapies as a complement to the conventional physiotherapy, to establish protocols and guidelines and to provide the best insight into the neurorehabilitation programs. Due to the COVID-19 pandemic, the hospitalization rate was reduced, as well as the rehabilitation program and hospital stay. For future research, we aim to recreate the study with a larger sample size, and a longer rehabilitation program during hospital stay, perhaps in correlation with a tele-rehabilitation strategy after discharge, as well. This would allow us track the progress and ensure continuity of the rehabilitation program for post-stroke patients with benefits in both the short and the long-term.

## Figures and Tables

**Table 1:** Therapeutic interventions and parameters for the control group and the experimental group.

	Control Group	Experimental Group
Treatment type	CP+sham rESWT+Prokin	CP+rESWT+Prokin
CP session length	1 h/day; 5 days/week; 2 weeks	1 h/day; 5 days/week; 2 weeks
rESWT session length	7 min/session; 1 session/week; 2 weeks	7 min/session; 1 session/week; 2 weeks
Visual feedback session length	20 min/day; 5 times/week; 2 weeks	20 min/day; 5 times/week; 2 weeks

Abbreviations: CP: conventional physiotherapy; rESWT: radial extracorporeal shock wave therapy; sham rESWT: sham radial extracorporeal shock wave therapy.

**Table 2:** Characteristics of participants at baseline, clinical, and stabilometric outcome measures.

	CG (Mean, SD)	EG (Mean, SD)	*p*-Value
Variables	*n*, 11	*n*, 12	
Age (years)	68.18 (11.51)	60.33 (11.5)	0.11 ^a^
Weight (kg)	77.50 (11.53)	75.32 (17.88)	0.72 ^a^
Height (cm)	174.09 (8.08)	171.16 (10.77)	0.46 ^a^
Time since stroke onset (months)	24.97 (34.17)	25.02 (39.23)	0.99 ^a^
Gender (M/F)	7/4	6/6	
Stroke type (Ischemic/Hemorrhagic)	8/3	9/3	0.5 ^c^
Affected side of the body (Right/Left)	6/5	4/8	0.87 ^c^
**Clinical outcome measures**			
MAS	2.54 (0.52)	2.58 (0.51)	0.86 ^a^
Knee PROM (degrees)	116.90 (5.16)	116 (4.78)	0.66 ^a^
Ankle PROM (degrees)	39.27 (3.16)	39.91 (4.1)	0.65 ^a^
VAS	3.09 (1.13)	3 (1.12)	0.84 ^a^
Clonus Score	2.36 (1.28)	2.25 (1.23)	0.82 ^a^
TIS	14.63 (2.33)	14.58 (2.71)	0.85 ^b^
Tinetti Assessment Tool	15.81 (4.33)	14.83 (5.62)	0.53 ^b^
FAC	4.72 (0.9)	4.58 (1.37)	1.00 ^b^
FMA-LE	19.27 (1.95)	19.66 (2.1)	0.60 ^b^
**Stabilometric outcome measures**			
Dynamic	4.74 (1.04)	5.27 (3.01)	0.57 ^a^
Trunk	202.54 (112.36)	193.21 (114.75)	0.84 ^a^
Limits of stability	46.82 (9.17)	52.23 (20.44)	0.41 ^a^
Static perimeter, mm (EO)	660.09 (289.16)	626.52 (244.94)	0.76 ^a^
Static ellipse area, mm^2^ (EO)	537.60 (95.62)	539.98 (276.06)	0.98 ^a^
Static perimeter, mm (EC)	1079.28 (558.89)	1147.16(492.65)	0.76 ^a^
Static ellipse area, mm^2^ (EC)	1092.48 (661.60)	1111.07 (467.81)	0.93 ^a^

Abbreviations: CG: control group; EC: eyes closed; EG: experimental group; EO: eyes open; FAC: Functional Ambulation Categories; FMA-LE: Fugl-Meyer Assessment for Lower Extremity; MAS: Modified Ashworth Scale; M/F: Male/Female ratio; PROM: passive range of motion; SD: standard deviation; TIS: Trunk Impairment Scale; VAS: Visual Analogue Scale. Differences between groups were calculated by Independent *t*-test ^a^, Mann–Whitney U test ^b^ or Pearson’s Chi-square test ^c^ depending on the data. *p*-value < 0.05.

**Table 3:** Comparison of change score of clinical outcome measures between CG and EG post-treatment.

Clinical Outcome Measures	CG (Mean, SD) *n* = 11 Post-Treatment	Change Score	EG (Mean, SD) *n* = 12 Post-Treatment	Change Score	Diff.	*p*-Value
MAS	2.18 (0.75)	0.36	1.50 (0.52)	1.08	0.72	0.02 ^a^
Knee PROM (degrees)	122.63 (5.4)	5.73	126.33 (3.96)	10.33	4.6	0.07 ^a^
Ankle PROM (degrees)	44.09 (3.47)	4.82	48.33 (2.26)	8.17	3.35	0.03 ^a^
VAS	2.09 (1.22)	1	1 (0.85)	2	1	0.02 ^a^
Clonus score	1.90 (1.13)	0.46	0.83 (0.83)	1.42	0.96	0.01 ^a^
TIS	17.54 (2.06)	2.91	18.66 (2.38)	4.08	1.17	0.2 ^a^
Tinetti Assessment Tool	21.09 (3.50)	5.28	24.66 (2.83)	9.83	4.55	0.02 ^b^
FAC	5.45 (0.82)	0.73	5.5 (0.79)	0.92	0.19	0.92 ^b^
FMA-LE	22.36 (2.06)	3.09	24.75 (2.01)	5.09	2	0.01 ^b^

Abbreviations: CG: control group; Diff: Difference of the change score; EG: experimental group; FAC: Functional Ambulation Categories; FMA-LE: Fugl-Meyer Assessment for Lower Extremity; MAS: Modified Ashworth Scale; PROM: passive range of motion; SD: standard deviation; TIS: Trunk Impairment Scale; VAS: Visual Analogue Scale. Differences between groups were calculated by Independent *t*-test ^a^ and Mann–Whitney U test ^b^ depending on the data. *p*-value < 0.05.

**Table 4:** Comparison of change score of stabilometric outcome measures between CG and EG post-treatment.

Stabilometric Outcome Measures	CG (Mean, SD) *n* = 11 Post-Treatment	Change Score	EG (Mean, SD) *n* = 12 Post-Treatment	Change Score	Diff.	*p*-Value
Dynamic	4.39 (0.86)	0.35	3.10 (1.66)	2.17	1.82	0.03
Trunk	311.58 (128.28)	109.04	458.85 (166.33)	265.64	156.6	0.02
Limits of stability	51.92 (7.76)	5.1	68.37 (19.12)	16.14	11.04	0.01
Static-perimeter, mm (EO)	624.52 (201.91)	35.57	424.48 (108.40)	202.04	166.47	0.01
Static-ellipse area, mm^2^ (EO)	482.81 (147.31)	54.79	328.59 (182.17)	211.39	156.60	0.03
Static-perimeter, mm (EC)	943.53 (412.42)	135.75	734.02 (332.75)	413.14	277.39	0.1
Static-ellipse area, mm^2^ (EC)	1021.83 (583.39)	70.65	609.77 (128.26)	501.30	430.65	0.04

Abbreviations: CG: control group; Diff: Difference of the change score; EC: eyes closed; EG: experimental group; EO: eyes open; SD: standard deviation. Differences between groups were calculated by means of the Independent *t*-test. *p*-value < 0.05.

## Data Availability

Datasets used and analyzed during the clinical trial are available from the corresponding author on reasonable request.
